# YEATS domain-containing 2 (YEATS2), targeted by microRNA miR-378a-5p, regulates growth and metastasis in head and neck squamous cell carcinoma

**DOI:** 10.1080/21655979.2021.1977553

**Published:** 2021-09-29

**Authors:** Tong Sha, Jia Li, Shiqun Sun, Jianing Li, Xuetao Zhao, Zehua Li, Zhi Cui

**Affiliations:** a The Third Department of Oral and Maxillofacial Surgery, Hospital of Stomatology, Jilin University, Changchun, People’s Republic of China; b Department of Oral and Maxillofacial Surgery Clinic, Hospital of Stomatology, Jilin University, Changchun, People’s Republic of China; c Department of Prosthodontics, Hospital of Stomatology, Jilin University, Changchun, People’s Republic of China; d Department of Endodontics, Hospital of Stomatology, Jilin University, Changchun, People’s Republic of China; e Department of Periodontics, Hospital of Stomatology, Jilin University, Changchun, People’s Republic of China; f Department of Pedodontics, Hospital of Stomatology, Jilin University, Changchun, People’s Republic of China

**Keywords:** YEATS2, miR-378a-5p, head and neck squamous cell carcinoma

## Abstract

Head and neck squamous cell carcinoma (HNSCC) is the sixth most common cancer worldwide with poor prognosis and the development of HNSCC is a complex process. Some research have found that YEATS domain-containing 2 (YEATS2) is highly expressed in non-small cell lung cancer and pancreatic cancer, whereas its function in HNSCC is left to be studied. The primary aim was to investigate the role of YEATS2 in proliferation, apoptosis, invasion and migration in HNSCC cells and explore the possible mechanisms. We found YEATS2 expression was elevated in HNSCC clinical samples. Our work also indicated YEATS2 knockdown inhibited cell proliferation, induced apoptosis, and diminished the migration and invasion capability in HNSCC cell lines, including Detroit562 and FaDu cells. Besides, these inhibiting effects of YEATS2 knockdown could be crippled by microRNA-378a-5p (miR-378a-5p) inhibitor. In conclusion, our data suggested that YEATS2 expression was regulated by miR-378a-5p and YEATS2 knockdown inhibited proliferation and metastasis while induced apoptosis in HNSCC cells.

## 1. Introduction

Head and neck squamous cell carcinoma (HNSCC), the sixth most common malignant tumor in the world, leads to more than 600,000 new cases and 380,000 deaths annually [1,2]. A large proportion of head and neck cancers can be classified into HNSCC, which contains squamous epithelium tumors originating in the oral cavity, oropharynx, larynx and hypopharynx [3]. Currently, tobacco, betel nut chewing, alcohol abuse and human papilloma virus (HPV) infection are the classical etiologic factors for HNSCC [4]. The standard interventions for HNSCC patients are surgical resections with or without adjuvant therapy, such as chemotherapy, radiation therapy and targeted therapy [5]. Tremendous efforts have been made in improving imaging modalities and surgical accuracy, increasing numbers of HNSCC patients receive curative surgical excision in clinical trials. However, the effectiveness of conventional treatments for locally advanced HNSCC patients is multimodal. Hence, it is essential to develop more efficient therapeutic strategy to manage HNSCC.

YEATS domain-containing 2 (YEATS2) protein is the scaffolding subunit for Ada-two-A-containing (ATAC) complex, which is identified as a conserved histone acetyltransferase associated with active transcription, stress signaling and mitotic progression [6–8]. Emerging evidence suggests that YEATS2 is a selective histone crotonylation reader by binding to histone H3 through crotonylation of lysine 27 [9,10]. It was confirmed that YEATS2 was the target gene of hypoxia-inducible factor 1α (HIF1α) and abundantly expressed in pancreatic cancer tissues, which was related to poor prognosis (Zeng et al., 2021). In addition, YEATS2 accelerated cell proliferation and migration in pancreatic cancer in vitro (Zeng et al., 2021). Similarly, Mi et al. also reported that YEATS2 level was observably amplified in non-small cell lung cancer (NSCLC) [11]. Meanwhile, their study suggested that YEATS2 knockdown whittled its interaction with acetylated histones, decreased ATAC complex-dependent promoter H3K9ac expression, and thereby deactivating gene expression essential for cell growth and apoptosis [11]. On the basis of the cancer genome atlas (TCGA) project, gene expression profiling interactive analysis website GEPIA (http://gepia.cancer-pku.cn/) and Ualcan (http://ualcan.path.uab.edu/analysis.html) prompt that YEATS2 expression is evidently elevated in HNSCC tissues compared with the non-cancer tissue, indicating YEATS2 may involve in the occurrence and development of HNSCC. Although YEATS2 was reported to accelerate cell proliferation, migration and invasion while suppress apoptosis in pancreatic cancer and NSCLC [Zeng et al., 2021; 11], whether YEATS2 could play an important part in HNSCC was left to be illustrated.

Currently, researchers have shown an increasing interest in microRNAs (miRNAs), a class of small non-coding RNAs containing 18 to 24 nucleotides [12]. It is well accepted that miRNAs modulate their target messenger RNA degradation or translation suppression at the post-transcriptional level by binding to the 3ʹ-untranslated region (3ʹ-UTR). Emerging evidence has suggested that miRNAs dysfunction may be one of the key causes for multiple diseases, especially cancer [13,14]. Besides, a variety of miRNAs, served as oncogenes or tumor suppressors, have been confirmed to be novel therapeutic targets in pre-clinic [15, Zeng et al., 2021]. For example, the recent study has found long non-coding RNA GAS5 (lncRNA GAS5) overexpression promotes cell apoptosis via regulating miR-378a-5p/SUFU axis in triple-negative breast cancer cells [16]. Moreover, miR-378a-5p acts as a tumor suppressor and is associated with the good prognosis in patients with renal cell carcinoma, melanoma and colorectal cancer [17, 18; 19]. Recently, Cui et al. has reported that the proliferation capacity is negatively regulated by miR-378a-5p expression in oral squamous cell carcinoma, the most frequent and aggressive HNSCC [20]. Besides, miR-378a-5p also inhibits angiogenesis through targeting Kallikrein-related peptidase 4 (KLK4) in oral squamous cell carcinoma cells [20]. It is interesting to note that the targeting relationship between miR-378a-5p and YEATS2 has been predicted by TargetScan (www.targetscan.org).

Inspired by the background and prediction, we preliminary infer that YEATS2 may involve in the regulation of proliferation, apoptosis, invasion and migration in HNSCC. Therefore, the main purpose of this work is to explore the effects of YEATS2 expression on the progress of proliferation, apoptosis, invasion and migration in HNSCC cells, and to investigate whether this effect is regulated by miR-378a-5p, which may provide new ideas for future treatment of HNSCC.

## 2. Materials and methods

### 2.1. Clinical tissue specimens

A total of 17 HNSCC patients diagnosed at Hospital of Stomatology, Jilin University were enrolled in this study. The clinical sample collection was carried out after the permit of the Ethical Committee of Hospital of Stomatology, Jilin University in accordance with the Declaration of Helsinki. Written informed consent was obtained from the HNSCC patients or their appropriate surrogates. The adjacent tissues far from the cancer tissue served as the control.

### 2.2. Cell culture and treatment

Human oral epithelial cell line HIOEC were purchased from Shanghai iCell Bioscience Inc. (China) and exclusively kept in an incubator at 37°C and 5%CO_2_. HNSCC cell line CAL-27 (Shanghai iCell Bioscience Inc., China) was grown in DMEM medium (Servicebio, Wuhan, China) with 10% fetal bovine serum (FBS, TIANHANG, Huzhou, China) in a humidified atmosphere of 37°C and 5%CO_2_. HNSCC cell line FaDu (Shanghai iCell Bioscience Inc., China), Detroit562 (Procell Life Science & Technology Co., Ltd., Wuhan, China) and UPCI-SCC-090 (Shanghai Zhong Qiao Xin Zhou Biotechnology Co., Ltd., China) were incubated in MEM medium (Servicebio, Wuhan, China) with 10% fetal bovine serum (FBS, TIANHANG, Huzhou, China) in a humidified atmosphere of 37°C and 5%CO_2_. The adherent Detroit562 and FaDu cells were exposed to YEATS2 siRNA and incubated at 37°C and 5%CO_2_ for indicated time frame before detection.

### 2.3. CCK-8 assay

Viability of Detroit562 and FaDu cells was detected at 0, 24, 48, 72 and 96 h after YEATS2 siRNA transfection using CCK-8 assay [21]. In short, the transfected cells were incubated in 10 μl cell counting kit-8 working solution for 2 h at 37°C according to the manufacturer’s instructions (Shanghai Beyotime Institute of Biotechnology, China). Finally, OD value at 450 nm was read by a microplate reader.

### 2.4. EdU assay

After transfection for 48 h, Detroit562 and FaDu cells were subjected to 10 μM EdU staining solution (KeyGEN BioTECH, China) for 2 h at 37°C. after that, the cells were fixed in 4% paraformaldehyde at room temperature for 15 min, permeabilized by 0.1% Triton X-100 at room temperature for 20 min, and incubated in Click-iT reaction mixture (KeyGEN BioTECH, China) in dark at room temperature for 30 min. 6-diamidino-2-phenylindole (DAPI, Biosharp, China) was used to stain the nuclei and the target fluorescence was visualized under fluorescence microscope at 400 × magnification.

### 2.5. TUNEL staining

The above treated cells were harvested and permeabilized by 0.1% Triton X-100 at room temperature for 15 min. Afterward, the abovementioned cells were subjected to the In Situ Cell Death Detection Kit (Roche, Switzerland) and all the experimental procedure was according to the manufacturer’s instruction. 6-diamidino-2-phenylindole (DAPI, Biosharp, China) was used to stain the nuclei. Finally, the target fluorescence was visualized under fluorescence microscope at 400 × magnification.

### 2.6. Apoptosis assay

The above mentioned Detroit562 and FaDu cells were harvested and resuspended in 500 μl binding buffer. Afterward, 5 μl Annexin V-fluorescein isothiocyanate and 5 μl propidiumIodide were added and incubated in dark for 15 min. Eventually, the stained cells were used to quantify the apoptosis rate by A flow cytometer (NovoCyte, USA).

### 2.7. Wound healing assay

According to the previous research with appropriate modification [22–24], the treated Detroit562 and FaDu cells were pre-incubated in Mitomycin C (Sigma, USA) at a concentration of 1 μg/mL for 1 h to inhibit proliferation before the wound healing assay. Then, the mono-layer Detroit562 and FaDu cells were wounded by a 200 μL pipette tip, kept in corresponding serum-free culture medium for another 24 h. Photographs at the tip wounded area were taken at 0 and 24 h under a microscope at 100 × magnification.

### 2.8. Transwell assay

The aforementioned Detroit562 and FaDu cells were seeded into the upper chambers of a 24-well plate. The chambers (Corning, USA) were coated with Matrigel (Corning, USA). The upper chambers were filled with serum-free culture medium while the lower chambers contained FBS. The Detroit562 and FaDu cells were grown in the upper chambers for 24 h to across the pre-coated Matrigel and migrated cells were stained with 0.5% crystal violet. Finally, the number of invasive cells was quantified under a microscope at 200 × magnification.

### 2.9. Dual-luciferase reporter assay

Bioinformatics prediction website TargetScan (www.targetscan.org) forecasted the binding relationship between miR-378a-5p and YEATS2 3ʹ-UTR. Detroit562 cells were co-transfected with YEATS2 3ʹ-UTR wild type (Wt) or YEATS2 3ʹ-UTR mutant type (Mut) reporter plasmid and miR-378a-5p mimics or control mimics with Lipofectamine 3000 (Invitrogen, USA). To quantify the Firefly and Renilla luciferase activity, the Dual-Luciferase assay kit (KeyGEN BioTECH, China) was used and all the procedures were all according to the manufacturer’s protocol. The data were presented as Firefly/Renilla luciferase activity.

### 2.10. MiR-378a-5p inhibitor transfection

To prove the regulate relationship between miR-378a-5p and YEATS2, Detroit562 cells were co-transfected with miR-378a-5p inhibitor and YEATS2 siRNA. Analogously, the adherent Detroit562 cells were exposed to miR-378a-5p inhibitor and YEATS2 siRNA, and incubated at 37°C and 5%CO_2_ for 48 h before detection.

### 2.11. Western blotting assay

The aforementioned Detroit562 and FaDu cells or clinical tissue specimens were harvested and lysed in the ice-cold mixture of radio immunoprecipitation assay (RIPA) lysis (Solarbio, China) and phenylmethanesulfonyl fluoride (PMSF, Solarbio, China). The protein concentration was determined by using the BCA assay kit (Solarbio, China). Equal amount of protein was separated by 8%, 11% and 15% sodium dodecyl sulfate polyacrylamide gel electrophoresis (SDS-PAGE) and transferred onto a polyvinylidene fluoride (PVDF) membrane that blocked by 5% skim milk for 1 h at room temperature. Then, the PVDF membranes were subjected to corresponding primary antibodies at 4°C overnight, anti-rabbit or anti-mouse horseradish peroxidase (HRP)-labeled secondary antibody for 1 h at 37°C, and chemiluminescence kit to visualize the protein. Finally, Gel-Pro-Analyzer was used to quantify the intensity of protein bands. The primary antibodies were listed as follows: Rabbit anti-YEATS2 (dilution: 1:1000, ABclonal, China), rabbit anti-proliferating cell nuclear antigen (PCNA, dilution: 1:1000, ABclonal, China), rabbit anti-pro/cleaved caspase-3 (dilution: 1:1000, CST, USA), rabbit anti-pro/cleaved poly-ADP-ribose polymerase (PARP, dilution: 1:1000, ABclonal, China), rabbit anti-matrix metallopeptidase-2 (MMP-2, dilution: 1:1000, Proteintech, China), rabbit anti-matrix metallopeptidase-9 (MMP-9, dilution: 1:1000, Proteintech, China).

### 2.12. Real-time polymerase chain reaction (RT-PCR)

The total RNA of clinical tissue specimens was extracted using total RNA isolation kit (Tiangen, China) to synthesize cDNA in accordance with the manufacturer’s instruction of miRNA First Strand cDNA Synthesis (Tailing Reaction) (Sangon Biotech, Shanghai, China). The expression level of miR-378a-5p in the clinical samples was determined by the Exicycler 96 System (Bioneer, Korea) in the present of Power Taq PCR MasterMix (Solarbio, China) and SYB Green (Solarbio, China). MiR-378a-5p expression was analyzed by 2^−ΔCT^ method.

### 2.13. Statistical analysis

The results were expressed as the means ± standard deviation (SD). The statistical difference between three or more groups was determined by one-way ANOVA followed by Tukey’s multiple comparison test using GraphPad Prism 8.0 (GraphPad Software, San Diego, CA, United States). The data from two groups was analyzed by paired *t* test. All the experiments were repeated for more than three times. P value less than 0.05 was considered to be statistically significant. All the experiments were skilled and blinded to the grouping.

## 3. Results

Based on the background, we assumed miR-378a-5p/YEATS2 signaling was related to the progression of HNSCC. Hence, we firstly investigated the role of YEATS2 in proliferation, apoptosis, invasion and migration in HNSCC cells, and verified the regulation of miR-378a-5p on YEATS2 in turn.

### 3.1. Expression of YEATS2 in HNSCC tissues and cancer cell lines

**Figure 1.**
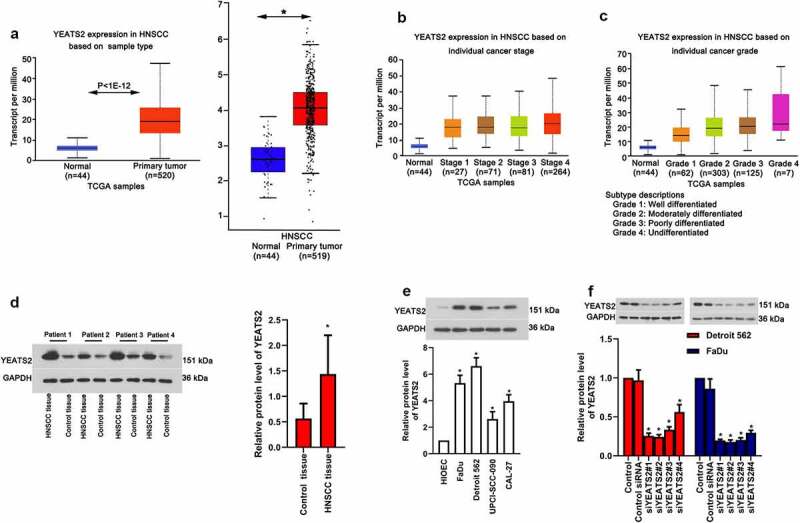
Expression of YEATS2 in HNSCC tissues and OSCC cell lines. (a) Expression of YEATS2 in HNSC tissues (Data cited from GEPIA and Ualcan website). (b) Expression of YEATS2 in HNSCC based on individual cancer stages. (c) Expression of YEATS2 in HNSC based on individual cancer grade (Data cited from Ualcan website). (d) Expression of YEATS2 in obtained clinical HNSCC samples. Data were represented as mean ± SD and analyzed by paired t test (n = 17). *p < 0.05 vs. Control tissue group. (e) Expression of YEATS2 in HIOEC, FaDu, Detroit562, UPCI-SCC-090 and CAL-27 cells. Data were represented as mean ± SD at least three independent experiments and analyzed by one-way analysis of variance (ANOVA) followed by Tukey’s multiple comparison test. *p < 0.05 vs. HIOEC cell group. (f) Western blot was used to detect YEATS2 siRNA transfection efficiency in Detroit562 and FaDu cells. Data were represented as mean ± SD at least three independent experiments and analyzed by one-way analysis of variance (ANOVA) followed by Tukey’s multiple comparison test. *p < 0.05 vs. the control group

As prompted by the data obtained from GEPIA (http://gepia.cancer-pku.cn/) and Ualcan (http://ualcan.path.uab.edu/analysis.html), YEATS2 expression was evidently elevated in HNSCC tissues (Figure 1(a), p < 0.05), which expression was also increased with advanced cancer stage and grade (Figure 1(b,c), p < 0.05). In accordance with these online data, our clinical results also found increased YEATS2 expression in HNSCC tissues (Figure 1(d), p < 0.05). To explore whether YEATS2 overexpression was related to HNSCC development, we firstly detected YEATS2 expression in human oral epithelial cell line HIOEC and different cancer cell lines, including FaDu, Detroit562, UPCI-SCC-090 and CAL-27. As exhibited in (Figure 1(e)), the expression of YEATS2 was relative abundant in Detroit562 and FaDu compared with other cancer cells (p < 0.05). Hence, YEATS2 was knocked down in Detroit562 and FaDu cells to inspect its effects (Figure 1(f), p < 0.05). The above results indicated that YEATS2 expression was up-regulated in HNSCC tissues and YEATS2 silence was attainable in HNSCC cell lines, Detroit562 and FaDu cells.

### 3.2. YEATS2 silence inhibited proliferation in Detroit562 and FaDu cells

**Figure 2.**
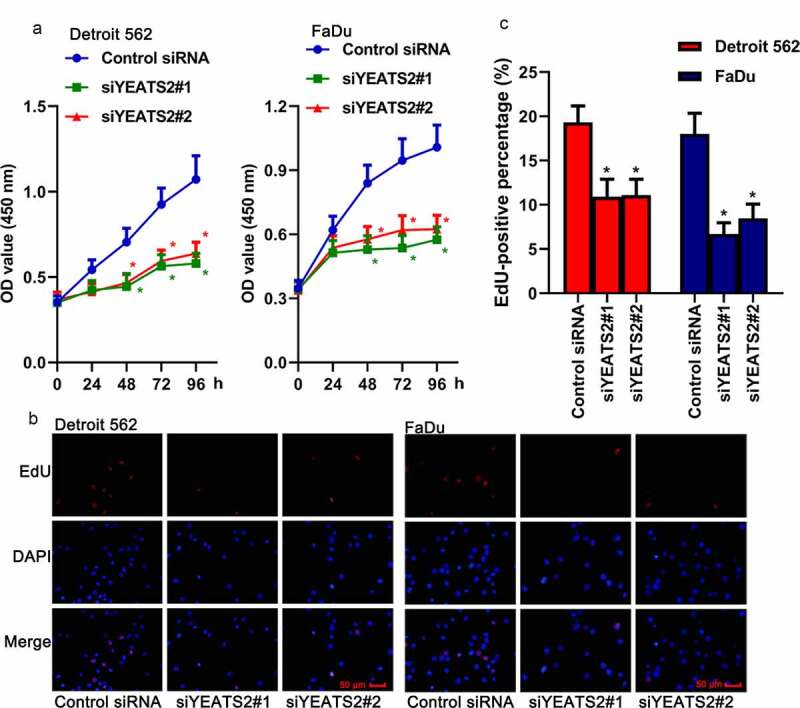
YEATS2 silence inhibited proliferation in Detroit562 and FaDu cells. (a) Cell viability was detected by CCK8 assay in Detroit562 and FaDu cells after YEATS2 siRNA transfection. (b) Proliferate ability was detected by EdU staining in Detroit562 and FaDu cells after YEATS2 siRNA transfection (at 400 × magnification). (c) Quantitative analysis for EdU staining. Data were represented as mean ± SD at least three independent experiments and analyzed by one-way analysis of variance (ANOVA) followed by Tukey’s multiple comparison test. *p < 0.05 vs. the control siRNA group

Afterward, the effects of YEATS2 expression on cell proliferation were analyzed. As described in (Figure 2(a)), CCK-8 assay showed that transfection with YEATS2 siRNA remarkably reduced cell viability compared with the control cells (p < 0.05). At the same time, EdU assay was also used to monitor cell proliferation. The fluorescence targeting EdU could be markedly reduced by YEATS2 siRNA transfection compared with the control one (Figure 2(b,c), p < 0.05). The above results suggested that YEATS2 silence was involved in proliferation suppression in Detroit562 and FaDu cells.

### 3.3. YEATS2 silence promoted apoptosis in Detroit562 and FaDu cells

**Figure 3.**
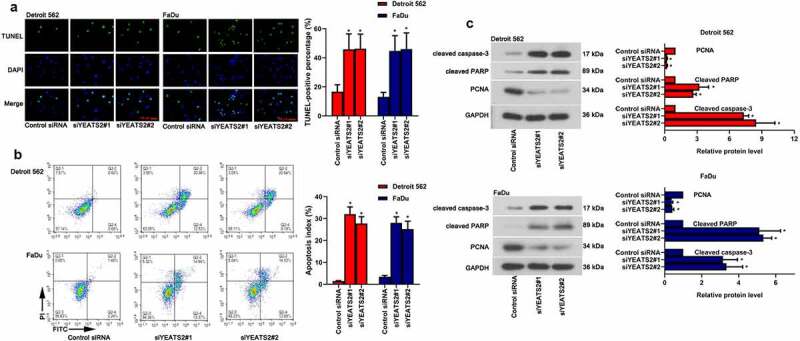
YEATS2 silence promoted apoptosis in Detroit562 and FaDu cells. Apoptosis was respectively detected by (a) TUNEL staining (at 400 × magnification) and (b) flow cytometry in Detroit562 and FaDu cells after YEATS2 siRNA transfection. (c) Western blot was used to analyze apoptosis-related protein cleaved caspase-3, cleaved PARP and PCNA expression in Detroit562 and FaDu cells after YEATS2 siRNA transfection. Data were represented as mean ± SD at least three independent experiments and analyzed by one-way analysis of variance (ANOVA) followed by Tukey’s multiple comparison test. *p < 0.05 vs. the control siRNA group

Except for abnormal proliferation, apoptosis is another key factor affecting oncogenesis and progression. As described in (Figure 3(a)), the number of TUNEL-positive cells was remarkably increased with YEATS2 siRNA transfection in Detroit562 and FaDu cells (p < 0.05). Consistent with the TUNEL staining results, YEATS2 siRNA transfection dramatically increased apoptosis rate in Detroit562 and FaDu cells (Figure 3(c), p < 0.05), which was confirmed by flow cytometer assay. Similarly, YEATS2 knockdown aggrandized the level of cleaved caspase-3 and cleaved PARP while suppressed PCNA expression in Detroit562 and FaDu cells (Figure 3(c), p < 0.05). The above results suggested that YEATS2 knockdown promoted apoptosis in Detroit562 and FaDu cells.

### 3.4. YEATS2 silence diminished migration and invasion ability in Detroit562 and FaDu cells

**Figure 4.**
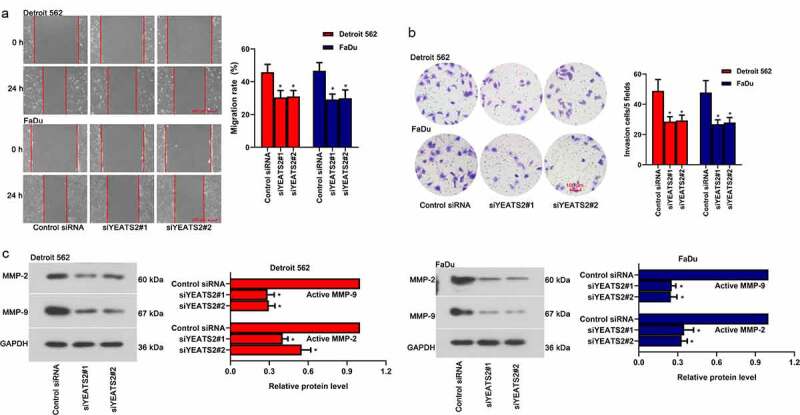
YEATS2 silence diminished migration and invasion ability in Detroit562 and FaDu cells. (a) Wound healing assay was conducted to detect the migration ability in Detroit562 and FaDu cells after YEATS2 siRNA transfection (at 100 × magnification). (b) Transwell assay was conducted to detect the invasion ability in Detroit562 and FaDu cells after YEATS2 siRNA transfection (at 200 × magnification). (c) Western blot was used to analyze active MMP-2 and active MMP-9 expression in Detroit562 and FaDu cells after YEATS2 siRNA transfection. Data were represented as mean ± SD at least three independent experiments and analyzed by one-way analysis of variance (ANOVA) followed by Tukey’s multiple comparison test. *p < 0.05 vs. the control siRNA group

To probe whether YEATS2 was one of the potential candidates regulating migration and invasion, which were involved in tumor progression. As described in (Figure 4(a,b)), inhibited migration and invasion ability were found in Detroit562 and FaDu cells after YEATS2 siRNA transfection (p < 0.05). Besides, the expression of active MMP-2 and active MMP-9 was decreased after YEATS2 silence in Detroit562 and FaDu cells (Figure 4(c), p < 0.05). The results indicated that YEATS2 knockdown suppressed migration and invasion in Detroit562 and FaDu cells.

### 3.5. miR-378a-5p regulated migration and invasion ability via YEATS2 in Detroit562 cells

**Figure 5.**
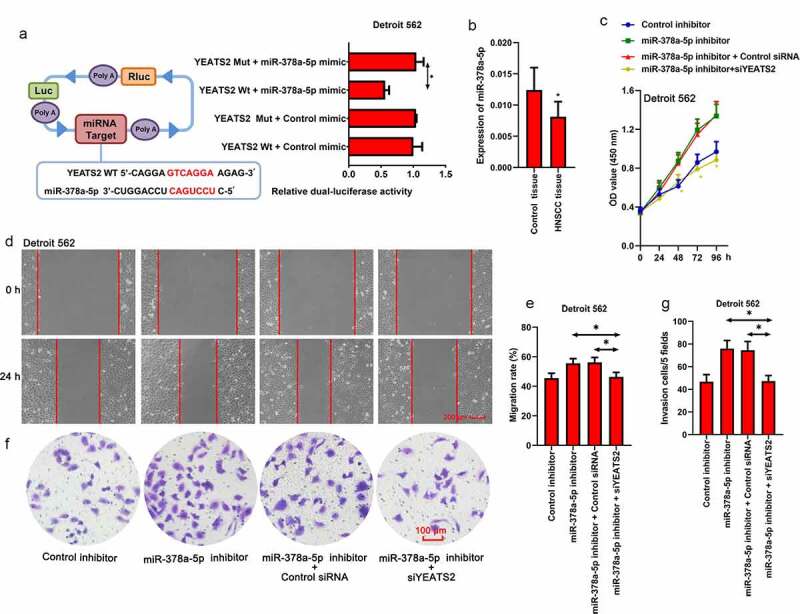
miR-378a-5p regulated migration and invasion ability via YEATS2 in Detroit562 cells. (a) Dual-Luciferase reporter assay was performed to detect the binding between miR-378a-5p and YEATS2 3ʹ-UTR in Detroit562 cells. (b) Expression of miR-378a-5p in obtained clinical HNSCC samples. Data were represented as mean ± SD and analyzed by paired t test (n = 17). *p < 0.05 vs. Control tissue group. (c) Cell viability was detected by CCK8 assay in Detroit562 cells after miR-378a-5p inhibitor and YEATS2 siRNA co-transfection. (d) and (e) Wound healing assay was conducted to detect the migration ability in Detroit562 cells after miR-378a-5p inhibitor and YEATS2 siRNA co-transfection (at 100 × magnification). (f) and (g) Transwell assay was conducted to detect the invasion ability in Detroit562 cells after miR-378a-5p inhibitor and YEATS2 siRNA co-transfection (at 200 × magnification). Data were represented as mean ± SD at least three independent experiments and analyzed by one-way analysis of variance (ANOVA) followed by Tukey’s multiple comparison test. *p < 0.05 vs. the indicated group

Since the potential binding relationship between miR-378a-5p and YEATS2 3ʹ-UTR was predicted by TargetScan (www.targetscan.org), we manipulated miR-378a-5p expression in Detroit562 cells. As shown in (Figure 5(a)), Dual-Luciferase reporter assay was implemented to verify the binding between miR-378a-5p and YEATS2 3ʹ-UTR. Besides, the decreased viability induced by YEATS2 silence could be diminished by miR-378a-5p inhibitor (Figure 5(c), p < 0.05). Simultaneously, the expression level of miR-378a-5p in HNSCC tissues was decreased compared with the paired control samples (Figure 5(b), p < 0.05). As described in (Figure 5(d-g)), the increased migration and invasion ability induced by miR-378a-5p inhibitor could be decreased by YEATS2 knockdown in Detroit562 cells (p < 0.05). These results suggested that miR-378a-5p was involved in the malignant phenotype via regulating YEATS2 expression in Detroit562 cells.

## 4. Discussion

In present work, we primarily explored the effects of YEATS2 expression on the progress of proliferation, apoptosis, invasion and migration in HNSCC cells, and preliminarily investigated whether miR-378a-5p/YEATS2 signaling was involved in the development process. Firstly, we found YEATS2 expression was increased in HNSCC tissues. Our present results suggested that YEATS2 knockdown inhibited cell proliferation, induced apoptosis, and diminished the migration and invasion capability in HNSCC cell lines, including Detroit562 and FaDu cells. Moreover, the oncogenic effects of miR-378a-5p inhibition could be crippled by YEATS2 silence in Detroit562 cells. In the meantime, the targeting relationship between miR-378a-5p and YEATS2 mRNA was also confirmed in our work. Collectedly, our present work revealed that YEATS2 depletion possessed tumor inhibitory function in HNSCC cells, which expression was regulated by miR-378a-5p.

Through bioinformatic analysis, we found YEATS2 was abnormally high expression in HNSCC tissues compared the non-cancer tissues. Hence, we preliminarily suspected YEATS2 overexpression might involve in HNSCC development. YEATS2 is the essential part of ATAC complex, which is closely related to transcription progress [8]. As a novel acetyllysine-binding module, the biological function of YEATS2 is remained to be studied. As mentioned in numerous studies, YEATS2 dysfunction could be found in the occurrence and development of various tumors, such as NSCLC and pancreatic cancer [Zeng et al, 2021; 11]. As reported by Zeng et al. YEATS2 protein was co-expressed and modulated by HIF1α via hypoxia response element, besides, the inhibitory function of HIF1α knockdown on proliferation and migration could be counteracted by YEATS2 overexpression in pancreatic cancer cells (Zeng et al, 2021). Similarly, YEATS2, a histone H3K27ac reader, served as an oncogene through regulating the transcriptional process in NSCLC tumorigenesis [11]. Firstly, our clinical results found increased YEATS2 expression in HNSCC tissues. Afterward, YEATS2 expression was detected in a series of HNSCC cancer cells, including FaDu, Detroit562, UPCI-SCC-090 and CAL-27. We found that YEATS2 was abundantly expressed compared with the normal human oral epithelial cell line, HIOEC cells. Since the expression of YEATS2 was relative higher in Detroit562 and FaDu cells than other cells, Detroit562 and FaDu cells were chosen for the future experiment. In order to investigate the function of YEATS2 expression on the tumor characteristics of HNSCC cells, we used YEATS2 specific siRNA to disturb YEATS2 expression in Detroit562 and FaDu cells. Both the mRNA and protein levels of YEATS2 were decreased, indicating YEATS2 silence was attainable in Detroit562 and FaDu cells, which laid the foundation for the future experiments. In line with the results of previous studies, we found YEATS2 silence suppressed cell proliferation while promoted apoptosis in Detroit562 and FaDu cells, preliminarily suggesting its carcinogenic function.

Tumor metastasis is another non-negligible point involved in tumor progression [25]. The previous research showed YEATS2 possessed the ability to regulate migration and invasion in pancreatic cancer in vitro [Zeng et al., 2021; 11]. Hence, the effects of YEATS2 on metastasis were determined in Detroit562 and FaDu cells. Fortunately, YEATS2 knockdown in Detroit562 and FaDu cells inhibited their ability to migrate and invade, which was in consistent with previous studies [Zeng et al., 2021; 11]. Matrix metallopeptidases (MMPs), the important proteolytic enzymes, possess the ability to degrade extracellular matrix components and basement membrane [26,27]. The important members, including MMP-2 and MMP-9, mainly work to decompose collagen IV in the basement membrane, break the tight and ordered junction between cells, and thereby contributing to tumor growth and metastasis [28,29]. As exhibited in the previous studies, decreased MMP-2 and MMP-9 expression has been shown to be associated with inhibited metastatic capacity in multiple human tumors, such as gastric cancer, colon cancer, ovarian cancer and HNSCC [30–32]. In the current study, we found MMP-2 and MMP-9 expression levels were markedly down-regulated by YEATS2 knockdown in Detroit562 and FaDu cells, which was accompanied by decreased migration and invasion ability. In this regard, the migration and invasion capacity in HNSCC cells was likely to be regulated by YEATS2.

Since numerous intracellular transduction cascades could regulate or be regulated by YEATS2, we only explored the one related to proliferation, apoptosis and metastasis. As predicted by TargetScan (www.targetscan.org), there was potential binding relationship between miR-378a-5p and YEATS2 3ʹ-UTR. At the same time, the anti-tumor effects of miR-378a-5p had been confirmed in a variety of tumors, such as renal cell carcinoma, colorectal cancer and oral squamous cell carcinoma [17,18,20]. Therefore, Dual-Luciferase reporter assay was implemented to verify the binding between miR-378a-5p and YEATS2 3ʹ-UTR. Fortunately, our data confirmed this target binding relationship. Additionally, the decreased viability as well as metastasis ability induced by YEATS2 silence could be diminished by miR-378a-5p inhibitor, suggesting the regulating effects of miR-378a-5p on YEATS2 in HNSCC cells. These results demonstrated that YEATS2 was a potential oncogene in HNSCC. Silencing YEATS2 suppressed cell proliferation, induced apoptosis, and inhibited migration and invasion in HNSCC cells. In addition, the anti-tumor effect of miR-378a-5p could be attributed to targeting YEATS2.

## Conclusion

YEATS2 expression of was elevated in HNSCC tissues. Down-regulating YEATS2 that was targeted by miR-378a-5p inhibited cell proliferation, induced apoptosis, and diminished the migration and invasion capability in Detroit562 and FaDu cells. Collectedly, the progress of proliferation, apoptosis, invasion and migration in HNSCC cells was regulated by miR-378a-5p/YEATS2 signaling.

## Data Availability

Some or all data, models, or code that support the findings of this study are available from the corresponding author upon reasonable request.

## Highlights

1. YEATS2 expression was elevated in HNSCC clinical samples.

2. YEATS2 knockdown inhibited cell proliferation and induced apoptosis in HNSCC cells.

3. YEATS2 knockdown diminished the migration and invasion capability in HNSCC cells.

4. YEATS2 was targeted by miR-378a-5p in HNSCC cells.
